# Risk factors for intimate partner violence in women in the Rakai Community Cohort Study, Uganda, from 2000 to 2009

**DOI:** 10.1186/1471-2458-13-566

**Published:** 2013-06-10

**Authors:** Fiona G Kouyoumdjian, Liviana M Calzavara, Susan J Bondy, Patricia O’Campo, David Serwadda, Fred Nalugoda, Joseph Kagaayi, Godfrey Kigozi, Maria Wawer, Ronald Gray

**Affiliations:** 1 Dalla Lana School of Public Health, University of Toronto, Toronto, Ontario Canada; 2 Makerere University, School of Public Health, Kampala, Uganda; 3 Rakai Health Sciences Program, Entebbe, Uganda; 4 Department of Epidemiology, Bloomberg School of Public Health, Johns Hopkins University, Baltimore, MD, USA

**Keywords:** Intimate Partner Violence, Risk Factors, Cohort, Women, Rakai, Uganda

## Abstract

**Background:**

Intimate partner violence (IPV) is a significant public health problem. There is a lack of data on IPV risk factors from longitudinal studies and from low and middle income countries. Identifying risk factors is needed to inform the design of appropriate IPV interventions.

**Methods:**

Data were from the Rakai Community Cohort Study annual surveys between 2000 and 2009. Female participants who had at least one sexual partner during this period and had data on IPV over the study period were included in analyses (N = 15081). Factors from childhood and early adulthood as well as contemporary factors were considered in separate models. Logistic regression was used to assess early risk factors for IPV during the study period. Longitudinal data analysis was used to assess contemporary risk factors in the past year for IPV in the current year, using a population-averaged multivariable logistic regression model.

**Results:**

Risk factors for IPV from childhood and early adulthood included sexual abuse in childhood or adolescence, earlier age at first sex, lower levels of education, and forced first sex. Contemporary risk factors included younger age, being married, relationships of shorter duration, having a partner who is the same age or younger, alcohol use before sex by women and by their partners, and thinking that violence is acceptable. HIV infection and pregnancy were not associated with an increased odds of IPV.

**Conclusions:**

Using longitudinal data, this study identified a number of risk factors for IPV. These findings are useful for the development of prevention strategies to prevent and mitigate IPV in women.

## Background

Violence against women is a serious and common human rights and public health problem, which causes significant morbidity and mortality worldwide [1]. Intimate partner violence (IPV) is one form of violence against women, and is defined as “behaviour within an intimate relationship that causes physical, sexual, or psychological harm, including acts of physical aggression, sexual coercion, psychological abuse and controlling behaviours” [2]. IPV is prevalent internationally; the WHO Multi-Country Study on Women’s Health and Domestic Violence identified a lifetime prevalence of physical and/or sexual partner violence ranging from 15% to 71% and a past year prevalence between 4% and 54% [3]. Estimates including psychological abuse and controlling behaviours would putatively be even higher.

Knowledge of context-specific risk factors for IPV is important to be able to appropriately focus prevention efforts. A recent World Health Organization (WHO) review noted the lack of data on IPV risk factors from longitudinal studies and from low and middle income countries [2]. This brings into question whether many of the factors identified to date are true risk factors, as opposed to variables which are associated with or consequences of violence, and also whether existing research findings are valid in low and middle income countries.

The WHO review identified risk factors for IPV and classified them into the four levels of an ecological framework: individual, relationship, community and societal levels factors [2]. Individual level factors are younger age, low socio-economic status, lower levels of education, separation or divorce, pregnancy, exposure to intra-parental violence in childhood, sexual abuse, depression, harmful use of alcohol or illicit drugs, acceptance of violence, and exposure to prior abuse or victimization. Relationship level factors are educational disparity, greater number of children, and marital dissatisfaction or discord. Community level factors are acceptance of traditional gender roles, unemployment, poverty, a high female illiteracy rate, acceptance of violence, a low proportion of women with high level of autonomy, a low proportion of women with higher education, and weak community sanctions (*i.e.* communities which lack legal sanctions and where women lack access to shelters and family support, and in which there is less moral pressure for neighbours to intervene if a woman is beaten). Societal level factors are divorce regulations, a lack of legislation on IPV within marriage, protective marriage laws, and traditional gender and social norms [2].

Recent cross-sectional studies of women in sub-Saharan Africa have identified some of these same risk factors for IPV [4-6], as well as substance abuse by a sexual partner [5,6], HIV infection [5,6], and having a male partner who has other sexual partners [6]. Two previous cross-sectional analyses of data from the Rakai Community Cohort Study (RCCS) from 1998 and 2001 revealed that younger age at first sex, shorter length of relationship, being in a consensual union (*i.e.* not legally or formally married) compared to being married, being married compared to having a boyfriend or other casual partner, having a partner who consumed alcohol before sex, and a woman perceiving her partner to have a higher risk of HIV infection were risk factors for IPV [7,8].

In this study, we aimed to identify risk factors for IPV in women of reproductive age in Rakai District, Uganda, using longitudinal data from seven survey rounds of the Rakai Community Cohort Study between 2000 and 2009. We assessed characteristics shown to be risk factors in other settings [2], as well as other putative risk factors [4-6].

## Methods

Since 1994, the Rakai Health Sciences Program has followed an open cohort of about 12,000 participants aged 15 to 49 years in 50 communities in the Rakai district of southwestern Uganda. The cohort has been described in detail elsewhere [9,10]. In brief, participants provide consent and are interviewed privately by interviewers of the same gender every 10 to 14 months, using a standardized questionnaire. Venous blood is collected for HIV-1 testing. More than 90% of eligible individuals participate in any given survey round.

Women who participated in the study between 2000 and 2009 were included in analyses if they reported at least one sexual partner during the study period and if they had provided any data on whether they experienced IPV during the study period.

Intimate partner violence (IPV) was defined as any physical, sexual, or verbal violence by a partner in an intimate relationship. Questions on IPV were modified from the Revised Conflict Tactics Scales (CTS2), and, in some analyses, type of IPV was classified as minor or severe as per the CTS2 [11,12]. Minor physical violence was defined as a husband or partner who had “pushed you, pulled you, slapped you or held you down,” and severe physical violence questions were having had a husband or partner who “punched you with a fist or with something that could hurt you,” “kicked you or dragged you,” “tried to strangle you or burn you,” or “attacked you with a knife, gun or other weapon.” Minor verbal violence was defined as a husband or partner having “verbally abused or shouted at you” and severe verbal violence was defined as a husband or partner who had “threatened you with a knife, gun, or other weapon.” Sexual violence was defined as a sexual partner having “used verbal threats to force you to have sex when you did not want to,” “physically forced you to have sex when you did not want to,” or “forced you to perform other sexual acts you did not want to do,” and all of these types of sexual violence were considered severe. Data were collected in all survey rounds on experiences of IPV in the current year, and in one survey round on experiences of all three forms of IPV ever. Data were collected on frequency of IPV in the past year for physical and verbal IPV in four survey rounds, and for sexual IPV in three survey rounds.

Potential risk factors for IPV were determined based on the literature and on which data were collected for the RCCS between 2000 and 2009. Since characteristics and experiences in early life may cause some of the characteristics and experiences in adulthood, and specifically, risk factors for IPV in adulthood may be on the causal pathway from early factors to IPV [13], risk variables were separated into early factors and contemporary factors, with early factors defined as variables that may affect women early in life (*i.e.* in childhood or in early adulthood) and contemporary factors defined as variables that may affect women in their current lives. These two groups of variables were analysed separately.

Early factors included sexual abuse in childhood or adolescence [6], age at first intercourse [14,15], whether first intercourse was coerced, and education level [4,6,15-22]. Childhood or adolescent sexual abuse was defined as having ever been sexually abused by a male before the age of 18, and was only asked about in one survey round. Age at first intercourse was taken from the round in which this question was first answered. Coerced first intercourse was defined as force having been used the first time a participant had sex, and was coded as yes if the participant indicated in any survey round that their first sex had been coerced. Education level was taken from the baseline survey round, *i.e.* from the first round of participation during the period under study.

Contemporary factors included demographic variables such as age [4,16-21,23-25], marital status [4,6,20-25], religion [16,19,22,25], occupation [4,21,25], partner’s occupation, and pregnancy status [5,20,23]; relationship variables such as type of relationship with partner [20], length of primary sexual partnership [20,24], difference in age between the participant and her partner [17,18,20,24,25], number of sexual partners in the past year [14-16,19,21,23,26-28], alcohol use before sex by the participant [14,18,26], alcohol use before sex by the participant’s partner [6,17,25], attitudes toward violence, and HIV status [4,6,15,17,20,21,23-25,29]. Pregnancy status was self-reported. A variable for attitudes toward violence was derived from a series of questions about whether a man is justified in beating his wife or partner in several situations, with acceptable defined as responding yes to any of these questions and not acceptable defined as responding no to all these questions, which were: she neglects household responsibilities, she disobeys the instructions of her husband/elders, she uses contraception without permission, she refuses her husband sex, he learns about his wife’s partner’s positive HIV serostatus, he learns about his positive HIV serostatus, argues over money, is unfaithful, or another reason. HIV status was defined as a positive result on two enzyme immunoassays, confirmed by Western blot or RT-PCR.

For participants who reported multiple partners in the past year, data about the partner with whom the participant reported having had sex most recently was used for the variables type of relationship with partner, alcohol use by partner, length of sexual partnership, and difference in age with partner; it was not possible to determine which specific partner (if any) had perpetrated violence.

### Statistical methods

The prevalence of IPV and of potential IPV risk factors was assessed. To identify early factors, logistic regression was used to estimate the bivariate and multivariable odds ratios (OR) and 95% confidence intervals (95% CI) associated with violence during the period of study participation. For contemporary factors, population-averaged logistic regression models were used to look at bivariate and multivariable associations [30], which account for repeated measures for each participant, using an exchangeable correlation matrix and a robust variance estimator, and modelling the associations between each variable and IPV in the subsequent year.

For each of early factors and contemporary factors, since there were multiple predictors of interest and to minimize the risk of Type I error of conventional backward selection models, an Allen-Cady modified backward selection procedure was used for the multivariable models [31]. Candidate variables were identified *a priori* as being of greater importance on the basis of known associations with violence, including sexual abuse in childhood or adolescence, coerced first sex, and education for early factors, and age, marital status, pregnancy status, difference in age with partner, use of alcohol, number of partners in past year, and attitudes toward violence for contemporary factors [2,27,32]. Additional variables hypothesized to be relevant were then ranked in order of putative importance, which in ascending order of importance for contemporary factors were relationship type, length of relationship, woman’s occupation, partner’s occupation, religion, and HIV status. For early variables, this included only age at first sex. Variables from the second group were deleted in order of ascending importance, *i.e.* age at first sex for early factors and beginning with relationship type for contemporary factors, until the first variable was encountered with a p value of p < 0.1, either by Wald test or by likelihood ratio test, depending on whether the variable was continuous, binary, or categorical.

Separate models were run to look at the associations between contemporary factors and risk of violence in the same year, in consideration of the fact that certain associations, such as the temporal association between pregnancy and violence, might not be adequately captured when looking at exposure and outcome data from sequential years. Analyses were done using Stata 12.

During the period under study, the Rakai Community Cohort Study was approved by institutional review boards at the Scientific and Ethics Committee of the Uganda Virus Research Institute, the Uganda National Council of Science and Technology, Columbia University, Johns Hopkins University, Johns Hopkins Bloomberg School of Public Health, and the Western. Ethics approval was obtained for this analysis from the University of Toronto.

## Results

Of the 20584 women who participated in the study over this period, 15081 (73.3%) were included in these analyses. One hundred twenty women were excluded because they were younger than 15 or older than 49 during the entire period under study, four women were excluded because they had a positive HIV test result and subsequent negative tests, 3228 women were excluded because they were not sexually active during the study period, and 2151 were excluded because they did not have any data on IPV during the study period.

IPV was common in this population, as shown in Table 1. Almost half of women experienced any violence during the study, with 41.4% reporting verbal violence, 31.3% reporting physical violence, and 30.0% reporting sexual violence. More than a quarter of women reported any IPV in the past year, with 23.2% experiencing verbal violence, 17.5% experiencing physical violence, and 15.1% experiencing sexual violence.

**Table 1:** Reported frequency of IPV by sexually active women in the Rakai Community Cohort Study, 2000 to 2009, N = 15081

**Type of IPV**	Past year,* n(%)	**Over study period, n(%)**
Any	4367 (29.0)	7504 (49.8)
Any sexual	2278 (15.1)	4528 (30.0)
Any verbal	3502 (23.2)	6250 (41.4)
Minor verbal	3489 (23.1)	6222 (41.3)
Severe verbal	250 (1.7)	893 (5.9)
Any physical	2637 (17.5)	4713 (31.3)
Minor physical	2531 (16.8)	4575 (30.3)
Severe physical	1115 (7.4)	2164 (14.3)

*Data from last survey round on which IPV data were available for each individual.

Most women (66%) who experienced any violence experienced more than one form of violence concurrently. In the most recent survey round of participation for the 4367 women who reported any IPV, 1064 women (24.4%) reported experiencing verbal violence only, 322 (7.4%) reported sexual violence only, and 94 (2.2%) reported physical violence only. Six hundred eighty-three women (15.6%) experienced verbal and physical violence only, 218 (5.0%) women reported verbal and sexual violence only, and 443 (10.1%) reported physical and sexual violence only, while 1289 women (29.5%) reported all three forms of violence.

### Early factors

Women commonly experienced violence early in life, with 31.3% of 1784 women reporting sexual abuse in childhood or adolescence and 17.5% of 11607 reporting coerced first sex, as shown in Table 2. The majority of women (67.9%) were between 15 and 19 years old at the time of first sex, with a mean age of 15.8 years, though more than a quarter were younger than 15.

**Table 2:** Frequency of early factors and bivariate and multivariable associations* from a logistic regression model of early factors and any IPV in sexually active women in the Rakai Community Cohort Study, 2000 to 2009, OR (95% CI), p value

**Early factor**		**n (%)**	**Bivariate association**		**Multivariable model 1: all early factors, OR (95% CI) N = 1370**	**Multivariable model 2: all early factors, adjusted for age at study baseline, OR (95% CI), N = 1370**
			**OR (95% CI)**	**N**		
Sexual abuse†	No	1225 (68.7)	1	1532	1	1
	Yes	559 (31.3)	1.57 (1.26, 1.96) p < 0.001		1.65 (1.29, 2.11) p < 0.001	1.68 (1.31, 2.15) p < 0.001
Education‡	<5 years	4604 (26.9)	1	9782	1	1
	5-7 years	7086 (41.3)	0.89 (0.81, 0.98) p = 0.02		0.92 (0.69, 1.22) p = 0.55	0.97 (0.73, 1.30) p = 0.86
	Secondary or higher	5456 (31.8)	0.61 (0.55, 0.68) p < 0.001		0.65 (0.48, 0.89) p = 0.008	0.68 (0.50, 0.93) p = 0.02
Age at first sex	Continuous	15.8 (2.2)§	0.92 (0.90, 0.94) p < 0.001	9219	0.96 (0.90, 1.01) p = 0.13	0.92 (0.87, 0.98) p = 0.01
Coerced first sex	No	9581 (82.6)	1	8134	1	1
	Yes	2026 (17.5)	4.17 (3.67, 4.76) p < 0.001		3.66 (2.54, 5.28) p < 0.001	3.54 (2.45, 5.12) p < 0.001

*From logistic regression models. †Sexual abuse in childhood or adolescence was only asked about in one of seven survey rounds. ‡Data on education are taken from the baseline survey round, *i.e.* the first survey round of participation during the period under study. §Mean (standard deviation).

In bivariate analyses, as shown in Table 2, sexual abuse in childhood or adolescence, coerced first sex prior to the study, and younger age at first intercourse were significantly associated with any IPV during the study, with a particularly strong association with coerced first sex (OR 3.66, 95% CI 2.54, 5.28). Women with secondary education or higher levels of education were significantly less likely to experience violence than women with less than five years of education. In a multivariable model including all early factors, associations were similar to those from bivariate models for each variable, except that age at first sex was no longer statistically significantly associated with IPV (p = 0.13). Removing age at first sex from the model resulted in a significant likelihood ratio test, as per the pre-specified criteria, so no variables were removed from the full model. A multivariable model controlling for age at study baseline had similar results to the multivariable model without age, except that age at first sex became statistically significantly associated with IPV, with an odds of 0.92 (95% CI 0.87, 0.98) per increase of one year of age.

### Contemporary factors

The frequency of characteristics and behaviours was examined, as shown in Table 3, using data from the baseline survey round. Almost thirty percent of women were in each of the age groups of 15 to 19, 20 to 24, and 25 to 34. Almost half of women were currently married and in a monogamous relationship, and most women were Catholic (59.0%), with a sizeable minority of Protestant (21.1%) and Muslim (15.8%) populations. The majority of women worked in agriculture (56.0%). More than a quarter of women reported that their partners worked in agriculture and another quarter reported that their partners worked in trade. About one fifth of women were pregnant (21.0%). Eighty-eight point six percent of women were in a relationship with a man who was older, and for almost half of these women, the man was five or more years older. Less than one in ten women reported having more than one partner in the past year (9.1%). More than one quarter of women reported using alcohol before sex (27.4%) and half of women reported that their partner used alcohol before sex (50.3%). The majority of women considered it would be acceptable for a man to beat his partner in at least one of a variety of situations (85.6%). Twelve point five percent of women were HIV-positive.

**Table 3:** Bivariate and multivariable associations* from a population-averaged logistic regression model of contemporary factors in the past year and any IPV in the current year in sexually active women in the Rakai Community Cohort Study, 2000 to 2009

**Contemporary factor**		**Baseline n (%)**	**Bivariate association**		**Multivariable model, 13533 observations, 6916 women**
			**OR (95% CI) p value**	**N in model†**	**OR (95% CI) p value**
Age	15-19	4861 (28.3)	-	-	-
	20-24	5018 (29.2)	-	-	-
	25-34	5008 (29.2)	-	-	-
	35-49	2285 (13.3)	-		-
	Continuous	28.3 (8.3)§	0.99 (0.99, 0.99) p < 0.001	29659, 10954	0.99 (0.98, 1.00) p = 0.01
Marital status	Never married	3777 (21.9)	1	29659, 10954	1
	Previously married	2287 (13.3)	1.35 (1.20, 1.52) p < 0.001		1.08 (0.85, 1.38) p = 0.53
	Currently married- polygamous	2611 (15.2)	1.75 (1.57, 1.95) p < 0.001		1.44 (0.92, 2.25) p = 0.11
	Currently married- monogamous	8562 (49.7)	1.83 (1.66, 2.01) p < 0.001		1.32 (0.85, 2.04) p = 0.22
Religion	Catholic	10115 (59.0)	1	29592, 10898	1
	Protestant	3613 (21.1)	0.99 (0.91, 1.07) p = 0.77		1.02 (0.92, 1.14) p = 0.66
	Muslim	2713 (15.8)	0.82 (0.75, 0.89) p < 0.001		0.88 (0.77, 1.02) p = 0.08
	Other	708 (4.1)	0.92 (0.78, 1.09) p = 0.34		1.00 (0.79, 1.28) p = 0.97
Occupation	Agriculture	9601 (56.0)	1	29658, 10953	1
	Shopkeeper/trading/vending	1510 (8.8)	0.87 (0.79, 0.95) p = 0.003		0.92 (0.80, 1.06) p = 0.25
	Housework	1334 (7.8)	1.02 (0.91, 1.16) p = 0.71		0.99 (0.80, 1.21) p = 0.90
	Professional	1171 (6.8)	0.70 (0.62, 0.80) p < 0.001		0.88 (0.73, 1.07) p = 0.19
	Student	1283 (7.5)	0.43 (0.36, 0.52) p < 0.001		1.04 (0.34, 3.15) p = 0.95
	Home brewing/bar worker/owner	444 (2.6)	1.26 (1.04, 1.52) p = 0.02		1.12 (0.84, 1.50) p = 0.45
	Other	1797 (10.5)	0.83 (0.76, 0.91) p < 0.001		1.01 (0.87, 1.17) p = 0.88
Partner’s occupation	Agriculture	4506 (27.4)	1	29171, 10682	1
	Shopkeeper/trading/vending	4409 (26.8)	0.95 (0.89, 1.01) p = 0.11		0.91 (0.82, 1.01) p = 0.07
	Professional	2028 (12.3)	0.81 (0.74, 0.89) p < 0.001		0.82 (0.71, 0.95) p = 0.01
	Student	485 (3.0)	0.44 (0.33, 0.59) p < 0.001		0.75 (0.26, 2.13) p = 0.58
	Home brewing/bar worker/owner	161 (1.0)	1.24 (0.98, 1.56) p = 0.08		1.13 (0.80, 1.60) p = 0.48
	Trucker	707 (4.3)	0.76 (0.65, 0.88) p < 0.001		0.82 (0.66, 1.03) p = 0.09
	Other	4169 (25.3)	0.98 (0.91, 1.05) p = 0.48		0.94 (0.84, 1.04) p = 0.22
Pregnancy status	No	10838 (79.0)	1	26263, 9734	1
	Yes	2877 (21.0)	1.11 (1.04, 1.18) p = 0.002		1.02 (0.92, 1.12) p = 0.71
Type of relationship	Husband	7502 (44.9)	1	29265, 10705	1
	Current consensual partner	3981 (23.8)	1.03 (0.97, 1.10) p = 0.35		0.88 (0.79, 0.67) p = 0.01
	Boyfriend	4941 (29.5)	0.69 (0.64, 0.74) p < 0.001		0.70 (0.47, 1.05) p = 0.09
	Other	301 (1.8)	0.78 (0.58, 1.04) p = 0.10		1.20 (0.48, 2.97) p = 0.70
Length of time in relationship	<3 years	5881 (43.4)	1	27050, 9956	1
	4-6 years	2774 (20.5)	0.94 (0.88, 1.01) p = 0.11		0.84 (0.75, 0.94) p = 0.002
	>6 years	4907 (36.2)	0.86 (0.80, 0.92) p < 0.001		0.79 (0.70, 0.90) p < 0.001
Partner age difference	Same age	991 (7.4)	1	23887, 9682	1
	≥10 years older	2098 (15.8)	1.10 (0.96, 1.26) p = 0.16		0.83 (0.69, 1.00) p = 0.05
	5-9 years older	3703 (27.8)	1.11 (0.98, 1.26) p = 0.11		0.83 (0.69, 0.99) p = 0.04
	<5 years older	5988 (45.0)	1.06 (0.94, 1.20) p = 0.34		0.83 (0.70, 0.99) p = 0.04
	<5 years younger	440 (3.3)	1.23 (1.02, 1.47) p = 0.03		1.02 (0.79, 1.32) p = 0.87
	≥5 years younger	97 (0.7)	1.30 (0.91, 1.86) p = 0.15		1.21 (0.76, 1.91) p = 0.42
Number of partners in past year	1	14653 (90.9)	1	27892, 10419	1
	>1	1473 (9.1)	1.26 (1.12, 1.42) p < 0.001		1.27 (0.99, 1.62) p = 0.06
Woman’s use of alcohol before sex	No	12086 (72.6)	1	29081, 10686	1
	Yes	4563 (27.4)	1.32 (1.25, 1.40) p < 0.001		1.21 (1.10, 1.32) p < 0.001
Partner’s use of alcohol before sex	No	7722 (49.7)	1	25525, 10381	1
	Yes	7826 (50.3)	1.48 (1.40, 1.56) p < 0.001		1.46 (1.34, 1.60) p < 0.001
Attitudes toward violence	Not acceptable	1551 (14.4)	1	22088, 9541	1
	Acceptable	9187 (85.6)	1.48 (1.36, 1.60) p < 0.001		1.43 (1.28, 1.59) p < 0.001
HIV status	Negative	14143 (87.5)	1	28677, 10620	1
	Positive	2013 (12.5)	1.02 (0.93, 1.13) p = 0.61		1.03 (0.90, 1.18) p = 0.65

*From repeated measures logistic regression models. †N in model is number of observations, number of groups (*i.e.* women).

Table 3 shows the results of bivariate and multivariable models of contemporary factors in the past year and any IPV in the current year. For the multivariable model, all variables were initially included, and all variables were retained in the final model since the likelihood ratio test was significant on removing relationship type, as per the specified criteria. Factors which were significantly positively associated with IPV in the multivariable model were use of alcohol before sex, having a partner who uses alcohol before sex, and thinking that a man beating his wife is acceptable. Factors that were significantly negatively associated with IPV were older age, having a partner who is a professional compared to a partner who works in agriculture, being in a relationship with a consensual partner compared with being in a relationship with a husband, being in a relationship lasting four or more years compared to a relationship of three or fewer years, and having a partner who is older compared to a partner the same age. Marital status, religion, number of partners in the past year, pregnancy status, and HIV status were not significantly associated with violence.

Multivariable models of the association between contemporary factors in the current year and IPV in the current year revealed qualitatively similar associations (data not shown). Of note, pregnancy was not a risk factor for violence in the same year, and in fact was associated with a lower risk of IPV with an odds ratio of 0.91 (95% CI 0.84, 0.98).

## Discussion and conclusion

As found in diverse international studies, IPV is prevalent in this population and most women who experienced IPV reported experiencing more than one form of violence concurrently. Several of the risk factors identified in this longitudinal study are consistent with existing evidence [2], including sexual abuse in childhood or adolescence, lower levels of education, forced first sex, younger age, alcohol use by women and by their partners, being in a relationship of shorter duration, and thinking that violence is acceptable. Similar to prior cross-sectional studies of RCCS data collected between 1998 and 2001 [7,8], this study also identified younger age at first sex as a risk factor. Additional risk factors for IPV identified in this study were coerced first sex and having a partner who is the same age or younger.

In contrast with much of the literature on risk factors for IPV [2], but consistent with another analysis of RCCS data [8] and other studies from sub-Saharan Africa [25,29], being pregnant was not associated with experiencing IPV in this study. There are several methodological factors which could lead to this inconsistency. First, longitudinal data were used in this study, so that the temporal sequence of pregnancy predating IPV was modelled appropriately. However, it was not possible to determine the exact timing of the pregnancy relative to the outcome of IPV, since data on the timing of the pregnancy were not collected. This is relevant since studies have found that the rates of IPV vary prior to, during, and subsequent to pregnancy [33,34], and so the lack of specificity in the modelling of the timing of pregnancy relative to IPV may preclude the identification of an association if one exists. Second, in these analyses, other important variables were controlled for, such as acceptance of violence, younger age, and difference in age between partners, and these variables might positively confound the association between pregnancy and violence. Third, the definition of IPV used in other studies, *e.g.* which types of IPV were measured, may affect estimates of association. In this study, data on physical, verbal, and sexual IPV were included, however, there were no data collected on controlling behaviours, which could affect the magnitude of association identified if controlling behaviours were associated with pregnancy independently of other forms of IPV. Finally, the finding of a lack of association could reflect effect modification on the basis of geographical or cultural contexts, *i.e.* that the association between pregnancy and IPV may only exist in certain contexts.

Another notable finding of this study is that HIV infection was not associated with IPV in the subsequent year, with an unadjusted odds ratio of 1.02 (95% CI 0.93, 1.13) and an adjusted odds ratio of 1.03 (95% CI 0.90, 1.18). In other population-based studies from East Africa, one cross-sectional study [23] and one prospective study [35] identified a positive association between IPV and HIV, however, other studies found an association only between certain types of IPV and HIV [25,36] and for prevalent but not incident HIV infection [20]. The lack of association between HIV status and IPV in this study may reflect how the exposure and outcome were defined, *i.e.* that prevalent and incident cases of HIV and IPV were included in the exposure and outcome, respectively, the types of IPV included as noted above, or the period in which the exposure and outcome were measured (*i.e.* one year), which may not reflect the relevant period of exposure for this association; the inclusion and control of relevant confounders; or that HIV leading to IPV is not a significant pathway in this population.

The strengths of this study are its large size, high participation rates, prospective design, the inclusion of several important variables as potential predictors, the separation of early and contemporary factors in analyses, and the focus on a low income country. There are several limitations to this study. Some risk factors which may be relevant were not included in the analyses because data were not available, such as number of children, income level, and the gap in income and education between partners, which may result in residual confounding of the associations between examined variables and IPV. Some variables may not specifically identify risky behaviour, for example the use of any alcohol before sex as opposed to heavy alcohol use or more general problematic alcohol consumption, which may dilute any association and bias the estimate of the effects of problematic alcohol use toward the null. The questions on IPV were modified from the CTS2, however, this version of the scale has not been validated in this population, which could contribute to measurement error. Also, as noted earlier, no data were collected on controlling behaviours as part of the definition of IPV, which could lead to an underestimate of associations. For contemporary factors, the exposure period was assumed to be the year prior to IPV, and for early factors, only IPV during the period under study was examined. In fact, the relevant exposure period for contemporary factors may be longer or shorter than a year, and for both early and contemporary factors, may take place closer to or further from any IPV incidents; these temporal relationships have not yet been well defined. Since we did not assess the role of early and contemporary factors together in one model, we are unable to determine whether contemporary factors are on the causal pathway between early factors and IPV, as hypothesized, or, in the event that contemporary factors are not in the causal pathway between early factors and IPV, whether early or contemporary factors are more strongly associated with experiencing IPV. Finally, it was not possible to discern whether data on violence were relevant to a specific partner for women who had more than one partner, however, this would not likely affect the validity of the results since consistently fewer than seven percent of women had more than one sexual partner in each survey round.

In summary, this analysis confirms that certain established risk factors from other settings are associated with IPV in this setting, *i.e.* sexual abuse in childhood or adolescence, lower levels of education, forced first sex, younger age, alcohol use by women and their partners, being in a relationship of shorter duration and thinking that violence is acceptable. The data also suggest that several hypothesized risk factors may not be associated with IPV, *i.e.* pregnancy and HIV positivity. Finally, the analysis also identifies novel risk factors for IPV in this setting, *i.e.* younger age at first sex, coerced first sex, and having a partner the same age or younger. These findings are likely generalizable to other rural areas in sub-Saharan Africa and potentially elsewhere, and have direct implications for public health action in terms of the primary and secondary prevention of IPV.

There are diverse approaches to addressing violence against women [2], and the risk factors identified in this study suggest the need to develop strategies at various levels, recognizing that primary prevention of violence has proved to be challenging. At the societal level, gender transformative programming and policies are needed to shift the norms and attitudes of communities and individuals with respect to violence, with the ultimate goal of changing the acceptance of violence and consequently the rates of violence. Promising strategies to address gender norms have already been implemented in this context [37] and elsewhere in sub-Saharan Africa [38]. At an individual level, specific steps can be taken to decrease a woman’s vulnerability to violence, which may include optimizing access to basic education and changing the built and social environments in which women and girls live to optimize their safety. Further, work can be done with male perpetrators or with males at high risk of perpetrating violence. More research is needed to determine the effectiveness of specific interventions, in particular the relative effectiveness of different approaches in the context of low and middle income countries [2].

## Competing interests

The authors declare that they have no competing interests.

## Authors’ contributions

FK developed the research question and protocol, conducted analyses, and drafted the manuscript. LC, SB, and PO contributed to the analysis and interpretation of data. RG and JK were involved in the conception and design of the study, acquisition of data, and analysis and interpretation of data. DS, FN, GK, and MW were involved in conception and design of the study, and acquisition of data. All authors revised the manuscript and approved the final draft.

## Pre-publication history

The pre-publication history for this paper can be accessed here:

http://www.biomedcentral.com/1471-2458/13/566/prepub
